# Description of the Anterior Cruciate Ligament Reconstruction Technique with Mini-Lemaire Type Anterolateral Tenodesis through a Single Femoral Tunnel

**DOI:** 10.1055/s-0044-1779326

**Published:** 2024-04-10

**Authors:** Ricardo Moro, Victor de Carvalho Thá, Vinícius Rossoni Ruedas, Roberto Tauchmann, Gustavo Meira Dantas, Mohty Domit Filho

**Affiliations:** 1 Serviço de Ortopedia e Traumatologia, Hospital São Lucas de Campo Largo, Campo Largo, PR, Brasil; 2 Serviço de Ortopedia e Traumatologia, Hospital do Rocio, Campo Largo, PR, Brasil; 3 Serviço de Ortopedia e Traumatologia, Centro Ortopédico Ingá, Maringá, PR, Brasil

**Keywords:** anterior cruciate ligament injuries, anterior cruciate ligament reconstruction, tenodesis

## Abstract

The anterior cruciate ligament (ACL) injury causes anteroposterior and rotational instability in the knee. Intra-articular reconstructions often fail to achieve satisfactory rotational control, leading to persistent complaints of instability and subjecting the neo-ligament to increased stress. Young patients with high athletic demands and grade 2 or 3 pivot-shift often have a higher risk of re-rupture after isolated ACL reconstruction. Over the years, various techniques have been developed to address such situations. Among the described techniques, one of the most commonly used is the modified or "mini-Lemaire" lateral extra-articular tenodesis. Biomechanical studies demonstrate the versatility of the technique due to its relatively isometric behavior in flexion angles of 0-60° when the graft is introduced deeply to the lateral collateral ligament. It offers the possibility of fixation at different anatomical positions on the lateral femoral condyle and at different degrees of flexion. The objective of this study is to describe an accessible, reproducible technique that relies on materials widely available in our environment.

## Introduction

Anterior cruciate ligament (ACL) injury causes anteroposterior and rotational instability of the knee. Isolated intra-articular reconstructions often do not achieve satisfactory rotational control, maintaining instability and imposing greater stress on the neoligament. Young patients, with high athletic demands, who practice sports with rotation on the knee and with grade 2 or 3 pivot-shift have a higher risk of re-rupture after isolated ACL reconstruction. 1 2 3

In order to reduce residual instability and re-rupture, lateral extra-articular reconstructions are performed concomitantly with ACL reconstruction. Among the techniques described, one of the most used is the modified Lemaire-type or “mini-Lemaire” lateral extra-articular tenodesis (LET). Biomechanical studies show great versatility of the technique due to its close to isometric stretching pattern between 0-60° when the iliotibial band (ITB) tape is passed deep to the fibular collateral ligament (FCL). This allows its fixation to the femur to be carried out at different degrees of flexion (0-60°). 3 4 Studies also demonstrate that due to the dynamic tensioning effect that the FCL imposes on the graft, its fixation can be done at different anatomical points in the metaphyseal region of the lateral femoral condyle, as long as it is proximal to the femoral insertion of the FCL, providing the same rotational control characteristics. 3 4 5

It is important to highlight that the mini-Lemaire fixation point is often close to the femoral fixation of the ACL graft, with the confluence of the tunnels or small bone wall between them being a concern when using an interference screw, which is very common in our country. 6 Due to these concerns, staples or anchors are often used for this purpose, which can cause irritation and pain due to the material protusion, requiring removal. 2 Therefore, a technique that avoids such difficulties is desirable.

The indications for performing LET as well as technical variations are constantly evolving. 1 2 7 The objective of the present work is to describe an accessible and reproducible technique for anterolateral extra-articular reconstruction of the mini-Lemaire type, using the same femoral tunnel as the ACL, and fixing them together with a single interference screw, minimizing the risk of tunnel confluence and providing adequate fixation of the grafts with less material.

The present study did not require approval by an ethics committee because the technique described is a modification of widely performed procedures.

## Description of the Technique

Conventional patient positioning for knee arthroscopy. After intra-articular procedures, the femoral tunnel described below is created and then the ACL tibial tunnel in the usual way.

### Lateral Incision

Make an incision of approximately 3cm on the lateral aspect of the knee, about 2cm proximal to Gerdy's tubercle, proceeding proximally along the lateral aspect of the distal femur, at the level of the lateral epicondyle. Variations in length and position may be necessary depending on associated procedures and the thickness of the adipose tissue.

Dissect the subcutaneous tissue to achieve adequate exposure of the ITB, from Gerdy's tubercle to approximately 5cm proximal to the lateral epicondyle.

### Graft Removal

Remove a strip from the posterior half of the iliotibial tract measuring approximately 1 × 8cm. Release the strip from lateral adhesions, taking care not to damage adjacent structures such as the joint capsule, lateral collateral ligament, capsulo-osseous layer of the IT band, while maintaining its insertion on the proximal tibia at Gerdy's tubercle.

**Fig. 1:**
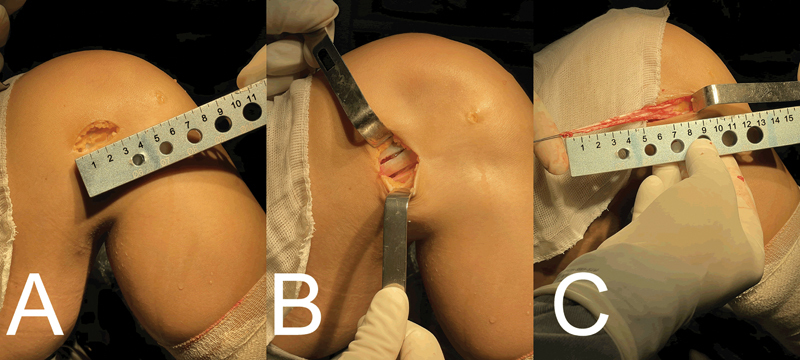
(( **A** ) Access with dissected ITB. ( **B** ) Demarcation of the graft donor area. ( **C** ) Graft removed and repaired.

Perform repair of the proximal stump using absorbable Vicryl suture size 1 ( Fig. 1 ).

### Femoral tunnel

Identify the FCL and lateral epicondyle by palpation through the gap of the previous ITB strip incision the previous incision to remove the ITB strip. If there is difficulty, perform varus with the knee in flexion to tension the ligament (figure “4” with lower limb).

**Fig. 2:**
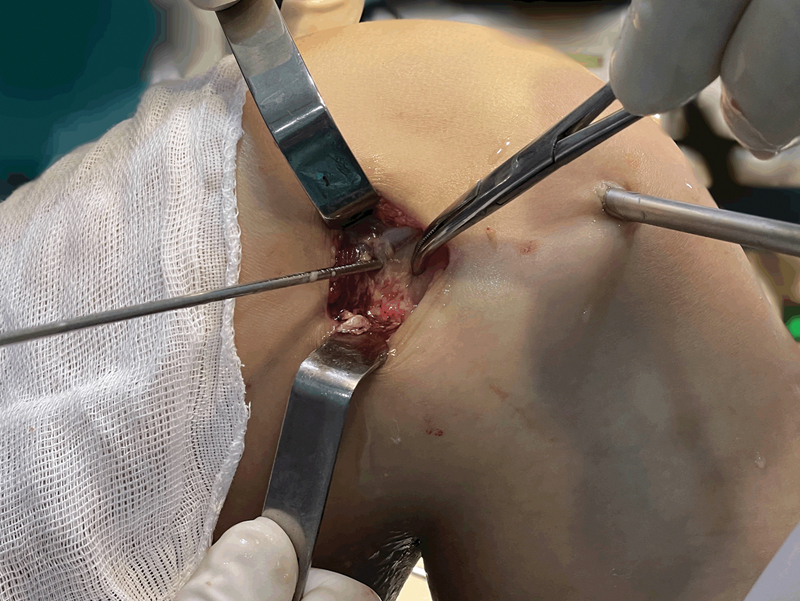
Identification of the lateral epicondyle (clamp) and femoral point of the mini-Lemaire (guide wire).

Pass the guide wire using the “outside-in” technique, ensuring that the wire entry point into the lateral femoral condyle is approximately 1 cm away from the lateral epicondyle ( Fig. 2 ).

The tunnel must be made with a drill with a diameter 1mm larger than the ACL graft, in order to allow joint passage of the ACL graft and the ITB strip into the tunnel.

Then drill the tibial tunnel in the usual way according to the size of the graft removed for the ACL.

### Deep Passage to the FCL

On the posterior and proximal margin of the FCL, make a small longitudinal incision with a scalpel to allow the introduction of a hemostatic clamp, taking care not to damage its fibers.

Insert the hemostatic clamp deep to the FCL through the incision made. It is possible to palpate the clamp immediately anterior to the FCL, where a new incision should be made to expose its tip. The clamp should always stay close to the ligament, being careful not to inadvertently damage the popliteal tendon or penetrate the intra-articular space.

**Fig. 3:**
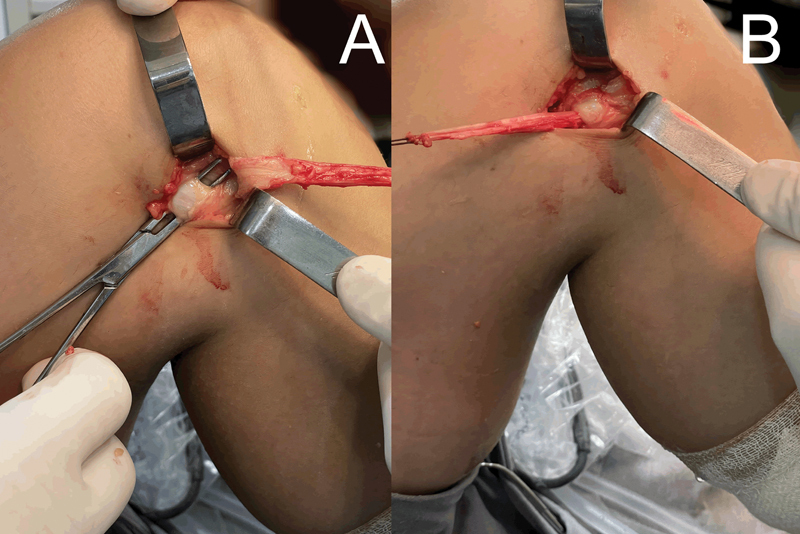
(( **A** ) Passage of the deep hemostat to the LCL. ( **B** ) Passage of the deep graft to the LCL.

After creating the tunnel deep to the FCL, pass the graft from anterior to posterior through it ( Fig. 3 ).

### Introduction of ACL and LET Grafts Through the Femoral Tunnel

After passing the LET graft deep into the LCL, use grasper forceps to pass the graft repair (Vicryl) through the femoral tunnel in order to visualize it in the intra-articular space. After, use the grasper again to expose the repair through the anteromedial portal. Then pull it so that the graft enters the tunnel.

Pass an Ethibond 5 thread through the femoral and tibial tunnels, where the ACL graft will be located, in the usual way.

**Fig. 4:**
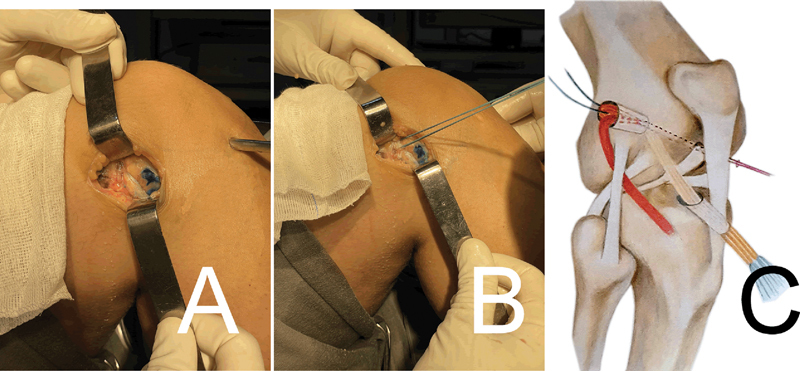
( **A** ) Passage of the LET in the femoral tunnel. ( **B** ) Passage of the flexor graft in the femoral tunnel. ( **C** ) Schematic drawing demonstrating position of grafts and tunnels.

During the introduction of the ACL graft, slight traction must be maintained on the ITB strip repair through its end in the anteromedial portal in order to prevent its extrusion through the external orifice of the femoral tunnel. Using the Ethibond 5 thread, introduce the ACL graft in a retrograde manner, first into the tibial tunnel and then into the femoral tunnel ( Fig. 4 ).

### Femoral Fixation of Grafts

The grafts are fixed to the femur with the knee in full extension.

Keep the knee in neutral varus/valgus and internal/external rotation.

**Fig. 5:**
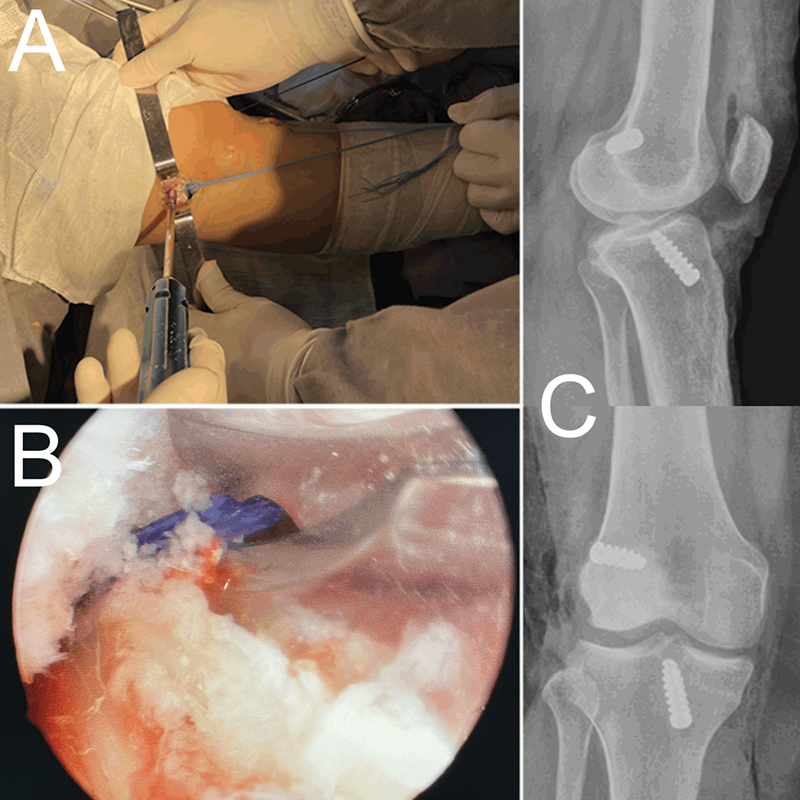
( **A** ) Panoramic photo with both grafts passed, position of fixation of the graft on the femur. ( **B** ) Photo of the intra-articular removal of Vicryl used to repair the TEL. It is possible to identify the ACL graft below the clamp to the left of the lateral wall of the intercondyle. ( **C** ) Post-operative X-ray of the described technique.

Maintaining gentle traction on the LET and ACL graft, fixation is performed with an interference screw ( Fig. 5 ).

Be careful not to over-tension the LET graft, in order to avoid increased pressure in the lateral compartment postoperatively. Do not leave a segment of the ACL protruding from the lateral femoral condyle, which could cause friction and subsequent pain.

### ACL Tibial Fixation

Fix the graft to the tibia in the usual way with an interference screw, maintaining distal traction on the graft, knee at 20-30°, neutral varus/valgus and internal/external rotation.

Remove a spare segment of the ACL graft from the tibia if necessary.

### Removal of Intra-articular Segment of LET

Remove the LET repair wire and, if necessary, a spare segment of the graft through arthroscopy with basket forceps ( Fig. 5 ).

### Iliotibial Band Closure

Suture the gap in the iliotibial tract corresponding to the donor area with interrupted stitches with Vicryl 1.

### Final Considerations

Studies indicate that any point proximal to the lateral epicondyle in the distal femoral metaphysis is suitable for mini-Lemaire reconstruction. A distance of 1cm is adopted, considering that ACL grafts should have a minimum diameter of 8mm, which can be larger. Thus, after drilling the ACL tunnel, a safe distance is preserved between the tunnel and the origin of the FCL. This distance can be increased if necessary.

Exposing the repair thread of the iliotibial tract through the anteromedial portal facilitates the retrograde passage of the ACL graft. After both are passed, the repair thread can be switched between portals as needed.

The decision is made to fix the mini-Lemaire graft on the femur with the knee in extension to ensure proper joint reduction and positioning of the graft in the distal femoral tunnel. This prevents joint malreduction, graft laxity, or over-tensioning, ensuring the maintenance of full knee extension.

It is not uncommon for the quadrupled hamstring graft (authors' preference) to have a greater length than the ACL tunnels. Retrograde introduction allows excess removal in the tibia without risking injury to the graft and facilitates tensioning. If anterograde introduction leads to excess graft in the femur, its removal may cause inadvertent injury to the graft or generate friction and pain if prominent.

If the described steps are followed, the occurrence of an intra-articular excess segment of the iliotibial tract at the end of the procedure is uncommon, requiring only the removal of the absorbable suture used at the femoral tunnel level in the intercondylar area. If there is an intra-articular segment of the ITB, it can be easily removed in the same way, taking care not to damage the ACL graft.

Postoperative rehabilitation is independent of ITB tenodesis and can be carried out in the usual manner for ACL reconstruction.
