# Supplementary material for: Description of the Anterior Cruciate Ligament Reconstruction Technique with Mini-Lemaire Type Anterolateral Tenodesis through a Single Femoral Tunnel
Source: Rev Bras Ortop (Sao Paulo). 2024 Apr 10;59(2):e313–7. doi: 10.1055/s-0044-1779326 (PMC11006511; doi:10.1055/s-0044-1779326)
Supplement: Related texts

# Research article: Descrição de técnica de reconstrução do ligamento cruzado anterior com tenodese ântero-lateral tipo mini-Lemaire através de túnel único femoral

**Keywords:** lesões do ligamento cruzado anterior, reconstrução do ligamento cruzado anterior, tenodese

## Abstract

A lesão do ligamento cruzado anterior (LCA) causa instabilidade anteroposterior e rotatória do joelho. Reconstruções isoladas intra-articulares muitas vezes não obtêm controle rotacional satisfatório, mantendo queixa de instabilidade e impondo ao neoligamento um maior estresse. Pacientes jovens, com alta demanda atlética e com pivot-shift grau 2 ou 3 apresentam um maior risco de rerrotura após reconstrução isolada do LCA. Ao longo dos anos diversas técnicas foram desenvolvidas na tentativa de mitigar tais situações. Dentre as técnicas descritas, uma das mais utilizadas é a Tenodese extra-articular Lateral tipo Lemaire modificada ou “mini-Lemaire”. Estudos biomecânicos demonstram versatilidade da técnica devido a seu comportamento relativamente isométrico em graus de flexão de 0-60° quando a fita é introduzida profundamente ao ligamento colateral lateral, com possibilidade de fixação em diferentes posições anatômicas no côndilo femoral lateral e em diferentes graus de flexão. O objetivo do estudo é descrever uma técnica acessível, reprodutível e dependente de material amplamente disponível em nosso meio.

## Introdução

A lesão do ligamento cruzado anterior (LCA) causa instabilidade anteroposterior e rotatória do joelho. Reconstruções isoladas intra-articulares muitas vezes não obtêm controle rotacional satisfatório, mantendo instabilidade e impondo ao neoligamento um maior estresse. Pacientes jovens, com alta demanda atlética, praticantes de esportes com rotação sobre joelho e com pivot-shift grau 2 ou 3 apresentam um maior risco de rerrotura após reconstrução isolada do LCA. 1 2 3

A fim de diminuir a instabilidade residual e rerrotura, realizam-se reconstruções extra-articulares laterais concomitantemente à reconstrução do LCA. Dentre as técnicas descritas, uma das mais utilizadas é a Tenodese extra-articular Lateral (TEL) tipo Lemaire modificada ou “mini-Lemaire”. Estudos biomecânicos mostram grande versatilidade da técnica devido ao seu padrão de alongamento próximo ao isométrico entre 0-60° quando a fita do trato iliotibial (TIT) é passada profundamente ao ligamento colateral fibular (LFC). Isso permite que a sua fixação no fêmur possa ser realizada em diferentes graus de flexão (0-60°). 3 4 Estudos demonstram ainda que devido ao efeito de tensionamento dinâmico que o LFC impõe ao enxerto, a fixação do mesmo pode ser feita em diferentes pontos anatômicos da região metafisária do côndilo femoral lateral, desde que este seja proximal a inserção femoral do LFC, proporcionando as mesmas características de controle rotacional. 3 4 5

É importante destacar que o ponto de fixação do mini-Lemaire muitas vezes fica próximo à fixação femoral do enxerto do LCA, sendo uma preocupação a confluência dos túneis ou pequena parede óssea entre eles quando usado parafuso de interferência, muito comum em nosso meio. 6 Devido a essas preocupações, muitas vezes utilizam-se grampos ou âncoras para tal finalidade, os quais podem gerar irritação e dor por saliência do material, com necessidade de retirada. 2 Dessa forma, é desejável uma técnica que evite tais dificuldades.

As indicações para realização da TEL assim como variações técnicas estão em constante evolução. 1 2 7 O objetivo do presente trabalho é descrever uma técnica acessível e reprodutível de reconstrução extra-articular anterolateral tipo mini-Lemaire, utilizando o mesmo túnel femoral do LCA e fixação conjunta dos mesmos com um único parafuso de interferência, minimizando o risco de confluência de túneis e proporcionando adequada fixação dos enxertos com menor quantidade de material.

O presente estudo dispensou aprovação por comitê de ética pois a técnica descrita é uma modificação de procedimentos amplamente executados.

## Descrição da Técnica

Posicionamento convencional do paciente para artroscopia de joelho. Após procedimentos intra-articulares, é realizado o túnel femoral descrito a seguir e, então, o túnel tibial do LCA de maneira usual.

## Incisão Lateral

Realizar incisão de aproximadamente 3cm em face lateral de joelho, cerca de 2cm proximal a tubérculo de Gerdy seguindo em direção proximal na face lateral do fêmur distal, ao nível do epicôndilo lateral. Variações em extensão e posição podem ser necessárias a depender de procedimentos associados e espessura de panículo adiposo.

Dissecar tecido subcutâneo até adequada exposição do ITB, do tubérculo de Gerdy até cerca de 5cm proximalmente ao epicôndilo lateral.

## Retirado do Enxerto

Retirar uma fita da metade posterior do trato iliotibial de cerca de 1 × 8cm. Liberar a fita de aderências laterais, tomando cuidado para não lesar estruturas adjacentes como a cápsula articular, ligamento colateral lateral, camada capsuloóssea do TIT, mantendo sua inserção na tíbia proximal no tubérculo de Gerdy.

Realizar reparo do coto proximal com fio absorvível de Vicryl 1 ( Fig. 1 ).

**Fig. 1:** ( **A** ) Acesso com TIT dissecado. ( **B** ) Demarcação da área doadora do enxerto. ( **C** ) Enxerto retirado e reparado.

## Túnel Femoral

Identificar por palpação o FCL e epicôndilo lateral através da lacuna da incisão anterior do ITB. Se houver dificuldade, realizar varo com joelho em flexão para tensionar o ligamento (figura de “4” com membro inferior).

Realizar a passagem do fio guia utilizando a técnica “de fora para dentro”, cuidando para que o ponto de entrada do fio no côndilo femoral lateral fique a cerca de 1cm de distância do epicôndilo lateral ( Fig. 2 ).

**Fig. 2:** Identificação do epicôndilo lateral (pinça) e ponto femoral do mini-lemaire (fio guia).

O túnel deve ser feito com broca de diâmetro 1mm maior que o enxerto do LCA, a fim de permitir passagem conjunta no túnel do enxerto do LCA e da fita do TIT.

Perfurar então o túnel tibial de maneira usual na medida do enxerto retirado para o LCA.

## Passagem Profunda ao FCL

Em margem posterior e proximal do FCL, realizar pequena incisão longitudinal com bisturi para permitir a introdução de pinça hemostática, com cuidado para não lesar suas fibras.

Introduzir pinça hemostática profundamente ao FCL na incisão realizada. É possível palpar a pinça imediatamente anterior ao FCL, onde deve ser realizada nova incisão para expor a sua ponta. A pinça deve ficar sempre rente ao ligamento, cuidando para não lesar inadvertidamente o tendão do poplíteo nem penetrar o espaço intra-articular.

Após realização do túnel profundamente ao FCL, o enxerto do trato iliotibial é passado de anterior para posterior ( Fig. 3 ).

**Fig. 3:** ( **A** ) Passagem da pinça hemostática profunda ao LCL. ( **B** ) Passagem do enxerto profundo ao LCL.

## Introdução dos Enxertos do LCA e TEL Através do Túnel Femoral

Após passagem do enxerto da TEL profundamente ao LCL, utilizar pinça grasper para passar o reparo do enxerto (Vicryl) através do túnel femoral de forma a visualizá-lo no espaço intra-articular. Após utilizar novamente a grasper para expor o reparo pelo portal anteromedial. Tracioná-lo então para que o enxerto entre no túnel.

Realizar a passagem de fio de Ethibond 5 através dos túneis femoral e tibial, onde ficará o enxerto do LCA, de maneira usual.

Durante a introdução do enxerto do LCA, deve-se manter leve tração no reparo da fita do TIT através de sua extremidade no portal anteromedial a fim de impedir sua extrusão pelo orifício externo do túnel femoral. Utilizando o fio de Ethibond 5, introduzir o enxerto do LCA de maneira retrógrada, primeiro no túnel tibial e após no túnel femoral ( Fig. 4 ).

**Fig. 4:** ( **A** ) Passagem do TEL no túnel femoral. ( **B** ) Passagem do enxerto de flexores no túnel femoral. ( **C** ) Desenho esquemático demonstrando posição de enxertos e túneis.

## Fixação Femoral dos Enxertos

A fixação dos enxertos no fêmur é feita com joelho em extensão completa.

Manter o joelho em neutro de varo/valgo e rotação interna/externa.

Mantendo uma gentil tração na LET e no enxerto do LCA, realiza-se fixação com parafuso de interferência ( Fig. 5 ).

**Fig. 5:** ( **A** ) Foto panorâmica com ambos os enxertos passados, posição de fixação do enxerto no fêmur. ( **B** Foto da remoção intra-articular do Vicryl que serve de reparo do TEL. É possível identificar abaixo da pinça o enxerto do LCA a esquerda a parede lateral do intercôndilo. ( **C** ) RX pós-operatório da técnica descrita.

Cuidado para não tensionar em excesso o enxerto do TEL, a fim de evitar aumento de pressão no compartimento lateral no pós-operatório. Não deixar segmento do LCA saliente no côndilo femoral lateral, o que poderá ocasionar atrito e dor subsequente.

## Fixação Tibial do LCA

Realizar fixação do enxerto na tíbia de maneira usual com parafuso de interferência, mantendo tração distal no enxerto, joelho em 20-30°, neutro de varo/valgo e de rotação interna/externa.

Retirar segmento sobressalente de enxerto do LCA na tíbia se necessário.

## Remoção de Segmento Intra-articular da TEL

Remover o fio de reparo da TEL e, se necessário, segmento sobressalente do enxerto através de artroscopia com pinças *basket* ( Fig. 5 ).

## Fechamento do Trato Iliotibial

Suturar a lacuna do trato iliotibial correspondente a área doadora com pontos interrompidos com Vicryl 1.

## Considerações Finais

Estudos indicam que qualquer ponto proximal ao epicôndilo lateral na metáfise do fêmur distal é adequado para a reconstrução do mini-Lemaire. Adota-se a distância de 1cm levando-se em consideração que enxertos do LCA devem ter no mínimo 8mm de diâmetro, podendo ser maiores. Assim, após perfuração do túnel do LCA, preserva-se distância segura entre túnel e origem do FCL. Tal distância pode ser maior se necessário.

A exposição do fio de reparo da fita do trato iliotibial através do portal anteromedial facilita a posterior passagem do enxerto do LCA de maneira retrógrada. Após a passagem de ambos, o fio de reparo pode ser trocado de portal de acordo com conveniência.

Opta-se por realizar a fixação do enxerto do mini-Lemaire no fêmur com joelho em extensão para assegurar adequada redução da articulação e posicionamento do enxerto na região distal do túnel femoral. Isso previne má redução da articulação, frouxidão ou sobre tensionamento do enxerto e assegura manutenção da extensão completa do joelho.

Não é incomum o enxerto quádruplo de flexores (preferência dos autores) ter maior comprimento que túneis do LCA. A introdução dele retrogradamente permite a remoção do excesso na tíbia sem risco de lesão do enxerto do TEL, além de facilitar o tensionamento. Caso a introdução seja anterógrada e haja enxerto sobressalente no fêmur, a remoção do mesmo pode causar lesão inadvertida na TEL ou gerar atrito e dor se saliente.

Se seguidos os passos descritos, é incomum a ocorrência de segmento sobressalente intra-articular da fita do trato iliotibial ao final do procedimento, necessitando apenas da remoção do fio absorvível utilizado ao nível do túnel femoral no intercôndilo. Caso haja segmento intra-articular do TIT, o mesmo pode ser removido com facilidade da mesma forma, cuidando para não lesar o enxerto do LCA.

A reabilitação pós-operatória independe da realização da TEL. Pode ser feita de maneira usual para LCA.
